# Acute Burn Treatment and History of Drug and Alcohol Addiction: Treatment Outcomes and Opioid Use

**DOI:** 10.3390/ebj3010002

**Published:** 2021-12-30

**Authors:** Eliana F. R. Duraes, Ya-Ching Hung, Mohammed Asif, Ashley Modica, Giulia Sikorski, Charles S. Hultman, Julie Caffrey

**Affiliations:** 1 Department of Plastic and Reconstructive Surgery—Burn Surgery, Johns Hopkins Bayview Medical Center, 4940 Eastern Avenue, Baltimore, MD 21224, USA; masif2@jhmi.edu (M.A.); amodica4@jhmi.edu (A.M.); grosano1@jhmi.edu (G.S.); chultma1@jhmi.edu (C.S.H.); jcaffre5@jhmi.edu (J.C.); 2 Department of Surgery, Sinai Hospital of Baltimore, 2435 Belvedere Avenue, Baltimore, MD 21215, USA; yhung@lifebridgehealth.org

**Keywords:** opioid crisis, drug addiction, burn injury

## Abstract

Treating pain in burn patients with a history of opioid or drug abuse is challenging. There is no consensus on pain management for burn patients with a history of drug usage. Our aim was to study the association of previous drug addiction and the treatment of acute burn patients, focusing on daily morphine milligram equivalent (MME) requirements and outcomes. We compared patients with (group 1) and without (group 2) a drug addiction history who were admitted to an American Burn Association verified burn center using the Premier database from 2013 to 2018 (n = 3046). Primary outcome was daily MME usage. Secondary outcomes included mortality, expected mortality rate, length of stay (LOS), and number of surgeries. Linear regression was performed to predict MME usage. In total, 16.6% of patients had history of drug abuse. In unadjusted analysis, group 1 had more males (68.1% vs. 57.3%, *p* < 0.001) and was younger (median 47 vs. median 53, *p* < 0.001) compared to group 2. In the adjusted analysis, group 1 required 84.1 additional daily MME usage than group 2 (*p* < 0.01). Drug addiction was associated with an increased number of surgeries, LOS, and higher daily MME usage. Patients with a history of drug usage required almost 60 mg of additional oxycodone per day.

## 1. Introduction

**Figure 1:**
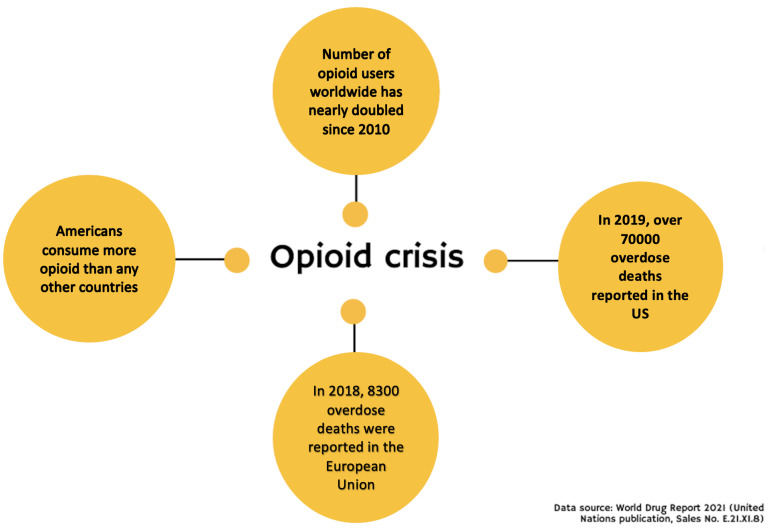
Opioid crisis.

Americans consume more opioids than any other country. Increased usage of opioids led this country to present the highest overdose death rate in the world [1] (Figure 1). Therefore, the US government declared the opioid crisis as a public health emergency in 2017 [2]. This pandemic is not limited to the US. In Europe, prescription opioids account for three-quarters of overdose deaths [3]. Data from Beirut and Lebanon also showed that opioids were the most common drugs for nonmedical usage among adolescents [3]. The opioid crisis is an extremely relevant topic to burn treatment as illicit drugs and alcohol abuse can be both a predisposing factor for the burn itself and an unwanted consequence of the burn treatment. According to Palmu et al., approximately 50% of adult burn patients were under the influence of alcohol at the time of the burn injury [4].

In general, treating pain in patients with a history of opioid usage or drug abuse is challenging [5]. The current available clinical recommendation is from expert panels [6]. However, pain management in this article is mostly related to post spinal surgery pain, which is very different from burn injury pain. Therefore, this recommendation understandably has limited application to burn patients. Furthermore, there is no consensus on pain management for burn patients with a history of drug usage. Meanwhile, the amount of narcotic prescription reportedly doubled over a seven-year period according to Tully in 2019 [7].

Independently of an illicit drug history, intravenous narcotics usage during surgical admissions has been shown to be associated with increased in-hospital complications, length of stay (LOS), and total inpatient hospital costs [8]. However, drug addiction is not included as a predictor for mortality rate in most of the commonly used mortality risk score systems [9]. Our hypothesis was that drug addiction could be a factor complicating outcomes of burn treatment. Therefore, this study assessed the amount of opioid usage between patients with and without a history of drug abuse and identified factors that predict the amount of daily morphine milligram equivalent (MME) for these two groups of patients. We specifically focused on burn treatment outcomes and measured additional (MME) requirement for adult burn patients with a history of drug abuse, compared to patients without a history of drug abuse.

Part of the data for this manuscript was published as an abstract in Journal of Burn Care & Research [10].

## 2. Methods

A retrospective review of patients who were admitted with the diagnosis of burn injury between 2013 and 2018 was performed using the Premier Database. The Premier Database is one of the largest US hospital-based databases that contains information on inpatient discharges and outpatient encounters. The dataset queried was from a single institution American Burn Association-verified burn center over a 5-year period of time.

Patients were identified using the International Classification of Diseases, Ninth and Tenth Revision (ICD-9 and ICD-10 codes) diagnosis codes for burn injuries (948.0–948.9 for ICD-9, and T20.0–T32.0 for ICD-10 codes). The admitting diagnosis codes for burn injuries were used in combination with specific codes for total body surface area (TBSA) burn (948.0–948.9 for ICD-9 codes, T31.0–T31.9 for ICD-10 codes). Drug abuse was defined as patients with a history of opioids abuse, heroin, cocaine, cannabis, inhalants, and alcoholism. The definition of drug abuse used was chosen to specifically include patients that had a history of drug abuse that is strong enough to be documented in the patient chart. Specific codes were used to identify drug abuse (303, 304.0–304.9, and 305.0–305.9 for ICD-9 codes; F10–F14, F17–F18, for ICD-10 codes).

Patient demographic data, such as age, race, ethnicity, types of drug abuse, TBSA, and length of intubation, were included. The primary outcome was MME usage. MME was calculated using the CDC MME calculator based on the medication patients received during their hospital stays [11]. Secondary outcomes included the actual mortality rate, expected mortality rate, LOS, and number of surgeries. The expected mortality was calculated using the Premier CareScience^TM^ Analytics, which takes into consideration comorbidities, principal diagnosis, procedures, type of admission (elective vs. emergency), travel distance to the hospital, admission source, discharge disposition, payer class, household income, race, and ethnicity. This expected mortality score is not specific for the burn patient. Patient demographic data was collected by a combination of self-report and utilization of the American Hospital Association Annual Survey Database. For most data elements in this dataset, less than one percent of patient records have missing information [12].

Descriptive analyses were performed using the Mann–Whitney U test and chi-square test. Multivariate analysis was performed using linear regression of daily MME usage adjusting for patient demographics, TBSA, and days intubated. Statistical analysis was performed with Stata SE statistical software, version 15.1 (StataCorp LP, College Station, TX, USA). The threshold for statistical significance was set at two-tailed *p* < 0.05. This study was approved by the Institutional Review Board of the hospital.

## 3. Results

A total of 3046 patients were studied. About one-sixth of patients had a history of drug abuse (n = 504, 16.6%). About one-third of patients had TBSA < 10% (36.5%). The most common substance identified was alcohol (49.4%), followed by opioid (34.7%), cannabis (24.4%), and cocaine (15.1%) (Table 1). Among the drug addiction group, 104 (20.7%) had a history of multiple substances abuse.

In the unadjusted analysis, patients who had a drug addiction were younger (median 47, IQR 33–57) compared to patients who were non-users (median 53, IQR 35–66, *p* < 0.001). They were also more likely to be males (68.1% vs. 57.3%, *p* < 0.001). Patients who had a history of drug abuse had a longer length of stay (median 6, IQR 3–13 vs. median 4, IQR 2–9, *p* < 0.001) and higher daily MME usage (median 61.4, IQR 20.5–119.1 vs. 33.8, IQR 7.5–72.9, *p* < 0.001). The groups showed similar Premier^®^ expected mortality rates (3.10% ± 9.07% vs. 3.56% ± 12.2%, *p* = 0.48). However, the actual mortality rate was significantly different between the two groups (2.0% for the drug abuse group vs. 3.1% for the non-users group, *p* = 0.01). There was no clinically relevant difference in TBSA between the groups (Table 2). 

In the adjusted analysis, patients who had a history of drug abuse significantly required 84.1 higher daily MME, compared to patients without a history of drug abuse (95% CI 68.9–99.3, *p* < 0.01). Increased age was also associated with lower daily MME usage (coefficient −1.1, *p* < 0.01) (Table 3). In addition, LOS and number of operations required during hospitalization were significantly higher for patients with a drug abuse history compared to patients who were not drug users (Table 4 and Table 5). Actual and expected mortality rates were not significantly different between the two groups after adjustment (*p* > 0.05, not shown in the table).

## 4. Discussion

Illicit drug and alcohol addiction have been an area of great concern for society in general, and the opioid crisis has escalated this problem and thus received more public interest. Drug and alcohol use in the burn population may interfere in all phases of burn care, from prevention to late sequelae after acute burn care [2]. This study showed additional 84.1 MME was given during admission for burn patients with a history of drug abuse, compared to patients without a history of drug abuse. This study also revealed that drug addiction was associated with an increased number of surgeries, LOS, and higher daily MME usage.

To our knowledge, our study is the first one to quantify additional MME usage among burn patients with a history of drug abuse. We demonstrate that patients with a history of drug abuse significantly required 84.1 higher daily MME, compared to patients without a history of drug abuse (95% CI 68.9–99.3, *p* < 0.01). In other words, patients who were substance users required almost 60 mg of additional oxycodone per day compared to patients who were non-users [11] This amount warrants additional attention for clinicians while prescribing opioids as the CDC recommended to avoid prescribing 90 MME or more opioid per day in patients with chronic pain [13]. This is due to studies showing that higher MME doses (MME 50–100) are significantly associated with a 1.9 to 4.6 higher risk of overdose [13]. In addition, a nationwide study has demonstrated that patients with higher MME usage (98 MME) are associated with higher rates of opioid overdose death, compared to patients with lower MME usage (47.7 MME) [14]. 

Our study result is consistent with the prior literature we have cited. We found that patients who have a history of drug abuse have a longer LOS and require more surgeries during hospitalization after adjustment for patient demographics. This is similar to the findings in Klifto et al. This large meta-analysis demonstrated that patients using substances were associated with a longer LOS and higher operation rates compared to patients who are non-users [15]. However, while Klifto et al. showed that patients using substances were associated with a higher mortality, our study did not show such a relationship on a regression model. This is similar to a recent publication by Williams et al., which also did not reveal such an association [16]. This may be due to the heterogeneous patient population included in Klifto et al., which included patients from rural and urban areas and patients of different ages. There is also a varying definition of drug abuse in their study, and this may be another reason for the different finding [15].

The mechanism for why drugs contribute to an increased LOS and increased number of operations is unclear. One possible reason is that illicit drug use may contribute to burn wound conversion. Alternatively, drug abuse may contribute to fire, which may cause increased rates of inhalation injury and ultimately, leading to intubation and prolonged hospital stay. However, the exact mechanism is beyond the scope of this study and will require further studies.

Our study has several strengths. First and foremost, our study design allowed us to tease out the true effect of drug abuse while adjusting for other confounders. Secondly, our definition of drug abuse allowed us to identify patients that had a true addiction, as our definition excludes patients that might receive opioids during transport or at the referring facility and therefore minimizes the confounding effect. However, this study also has some limitations. The duration and severity of drug abuse are unknown. It is also unclear whether patients had adequate pain control despite higher MME usage. Our study did not evaluate the mechanism of burn injury and thus cannot evaluate the causal relationship between drug abuse and burn injury. Finally, our study only included patients from one institution and therefore might not be representative of the nation. Our study is also limited to a 5-year period from 2013–2018 and therefore long-term and larger studies are needed to determine the impact of drug abuse on the burn population.

Guidelines for pain management for patients suffering from burn injury are limited. A nationwide study from 2007–2017 showed that overall opioid prescription has declined in the past decade, but there is significant variation among regions and age groups [17]. Tully et al. showed that the amount of discharge narcotic prescription in 2015 doubled when compared to those discharged in 2008 in a single burn center [1], even after controlling for age, burn size, intensive care unit stay, discharge day, substance abuse, comorbidity status, insurance, language, race, and ethnicity [7]. Although this discrepancy could be due to a historical time of under-treatment of pain, it does raises concerns and highlights the importance of establishing protocols to safely and efficiently control pain across different burn centers.

As a result, the American Burn Association published guidelines attempting to provide clinician treatment of acute pain management. The American Burn Association guideline is one of the recommended guidelines as some other international guidelines were found to be suboptimal in guiding pain management [18]. However, the guidelines still concluded that current available studies are inadequate to provide standard of care for pain control [19]. The literature has demonstrated that there is a lack of clinical trials evaluating the efficacy of medication in treating burn-induced pain [20]. Therefore, our study highlights future needs for investigating pain management for burn patients with a history of drug usage at the nation level. Our study offers data demonstrating current practice in pain management for burn patients with a drug abuse history and may be helpful in future guideline development.

Our study has several implications. We found that patients with a history of drug addiction was associated with an increased LOS. Median LOS for patients with a history of drug abuse and higher TBSA (>20%) was 28 days (data not shown in the table). Therefore, the lengthy admissions during acute burn treatment may serve as an opportunity for addiction treatments. Furthermore, the additional MME quantified in this study may help to prevent opioid overdose in patients with acute burn injury. CDC recommended to avoid prescribing 90 MME or more opioid per day in patients with chronic pain [13]. Our adjusted analysis showed patients with a history of drug abuse will require additional 84.1 MME, compared to patients without a history of drug abuse. This amount is very close to the amount of opioid that CDC recommended avoiding in their recommendation in order to prevent opioid overdose. Therefore, our study may help clinicians to address pain in the treatment of burn patients with a history of drug abuse while avoiding opioid-related complications.

## 5. Conclusions

Drug addiction is associated with an increased number of surgeries, LOS, and a higher daily MME usage after adjustment for confounders. Patients with a history of drug abuse significantly required 84.1 higher daily MME, which is almost 60 mg of additional oxycodone per day, compared to patients without a history of drug abuse. As studies have shown that higher MME usage is associated with opioid overdose, this amount of additional MME usage warrants additional attention for clinicians while treating patients with a history of drug abuse.

## Figures and Tables

**Table 1:** Patient demographics.

Variable	n (%) Median (IQR)
Age	52.0 (35.0, 64.0)
Male	1799 (59.1%)
TBSA	
<10%	1112 (36.5%)
10–20%	231 (7.6%)
20–30%	76 (2.5%)
30–40%	25 (0.8%)
40–50%	24 (0.8%)
50–60%	11 (0.4%)
60–70%	16 (0.5%)
70–80%	9 (0.3%)
80–90%	2 (0.1%)
>90%	5 (0.2%)
Drug addiction	504 (16.6%)
Alcohol	249 (49.4%)
Opioid	175 (34.7%)
Cannabis	123 (24.4%)
Sedative	10 (2.0%)
Cocaine	76 (15.1%)
Inhalant	5 (1.0%)
Days Intubated	<0.01 (<0.01, <0.01)
LOS	4.0 (2.0, 10.0)
Daily MME	37.7 (8.6, 79.3)

**Table 2:** Comparison between patients with drug addictions and non-users.

Variable	No Drug Addiction (n = 2542)	Drug Addiction (n = 504)	*p* Value
Age	53.0 (35.0, 66.0)	47.0 (33.0, 57.0)	<0.001
Male	1456 (57.3%)	343 (68.1%)	<0.001
TBSA			
<10%	937 (36.9%)	175 (34.7%)	0.013
10–20%	177 (7.0%)	54 (10.7%)	
20–30%	59 (2.3%)	17 (3.4%)	
30–40%	18 (0.7%)	7 (1.4%)	
40–50%	20 (0.8%)	4 (0.8%)	
50–60%	7 (0.3%)	4 (0.8%)	
60–70%	11 (0.4%)	5 (1.0%)	
70–80%	8 (0.3%)	1 (0.2%)	
80–90%	1 (<1%)	1 (0.2%)	
>90%	5 (0.2%)	0 (0.0%)	
Days Intubated	<0.01 (<0.01, <0.01)	<0.01 (<0.01, <0.01)	<0.001
LOS	4.0 (2.0, 9.0)	6.0 (3.0, 13.0)	<0.001
Daily MME	33.8 (7.5, 72.9)	61.4 (20.5, 119.1)	<0.001

**Table 3:** Adjusted analysis of daily MME usage.

Variable	Coef.	95 % CI		*p* Value
Age	−1.10	−1.41	−0.79	<0.01
Female (ref: male)	7.67	−3.91	19.24	0.19
TBSA				
<10%	1.31	−11.15	13.77	0.84
10–20%	−5.27	−27.26	16.71	0.64
20–30%	−13.32	−49.70	23.05	0.47
30–40%	−11.62	−75.64	52.39	0.72
40–50%	2.37	−63.00	67.74	0.94
50–60%	−24.72	−121.13	71.69	0.62
60–70%	120.86	39.36	202.35	<0.01
70–80%	−28.09	−131.87	75.68	0.60
80–90%	−50.52	−269.53	168.48	0.65
>90%	−36.94	−176.81	102.94	0.61
Drug addiction (ref: no)	84.13	68.92	99.33	<0.01
Days Intubated	0.18	−0.62	0.99	0.65

**Table 4:** Adjusted analysis of length of stay.

Variable	Coef.	95 % CI		*p* Value
Age	0.00	−0.02	0.03	0.90
Female (ref: male)	−0.22	−1.14	0.70	0.64
TBSA				
<10%	−2.11	−3.10	−1.12	<0.01
10–20%	1.20	−0.55	2.94	0.18
20–30%	6.73	3.84	9.62	<0.01
30–40%	-2.23	−7.32	2.85	0.39
40–50%	3.25	−1.95	8.44	0.22
50–60%	−2.04	−9.70	5.62	0.60
60–70%	12.78	6.31	19.26	<0.01
70–80%	12.28	4.03	20.52	<0.01
80–90%	−32.62	−50.02	−15.22	<0.01
>90%	−18.95	−30.06	−7.84	<0.01
Drug addiction (ref: no)	3.24	2.03	4.44	<0.01
Days Intubated	1.73	1.66	1.79	<0.01

**Table 5:** Adjusted analysis of number of OR visits.

Variable	Coef.	95 % CI		*p* Value
Age	<0.01	−0.01	<0.01	<0.01
Female (ref: male)	0.04	−0.06	0.14	0.42
TBSA				
<10%	−0.27	−0.38	−0.16	0.00
10–20%	−0.15	−0.33	0.04	0.13
20–30%	0.44	0.14	0.75	0.01
30–40%	0.52	−0.02	1.06	0.06
40–50%	1.11	0.56	1.67	<0.01
50–60%	2.46	1.65	3.28	<0.01
60–70%	4.66	3.97	5.35	<0.01
70–80%	−0.62	−1.49	0.26	0.17
80–90%	0.46	−1.39	2.31	0.63
>90%	−0.04	−1.23	1.14	0.94
Drug addiction (ref: no)	0.26	0.14	0.39	<0.01
Days Intubated	0.15	0.15	0.16	<0.01

## Data Availability

Premier Applied Sciences^®^ PI. Premier Healthcare Database White Paper: Data that Informs and Performs. Published 2020. Available online: https://learn.premierinc.com/white-papers/premier-healthcare-database-whitepaper (accessed on 11 October 2021).
